# Implementation of an Integrated Dielectrophoretic and Magnetophoretic Microfluidic Chip for CTC Isolation

**DOI:** 10.3390/bios12090757

**Published:** 2022-09-14

**Authors:** Kai Zhao, Penglu Zhao, Jianhong Dong, Yunman Wei, Bin Chen, Yanjuan Wang, Xinxiang Pan, Junsheng Wang

**Affiliations:** 1 Liaoning Key Laboratory of Marine Sensing and Intelligent Detection, Dalian Maritime University, Dalian 116026, China; 2 Department of Information Science and Technology, Dalian Maritime University, Dalian 116026, China; 3 Software Institute, Dalian Jiaotong University, Dalian 116028, China; 4 Department of Maritime, Guangdong Ocean University, Zhanjiang 524000, China

**Keywords:** dielectrophoresis, CTC separation, magnetophoresis, microfluidic chip

## Abstract

Identification of circulating tumor cells (CTCs) from a majority of various cell pools has been an appealing topic for diagnostic purposes. This study numerically demonstrates the isolation of CTCs from blood cells by the combination of dielectrophoresis and magnetophoresis in a microfluidic chip. Taking advantage of the label-free property, the separation of red blood cells, platelets, T cells, HT-29, and MDA-231 was conducted in the microchannel. By using the ferromagnet structure with double segments and a relatively shorter distance in between, a strong gradient of the magnetic field, i.e., sufficiently large MAP forces acting on the cells, can be generated, leading to a high separation resolution. In order to generate strong DEP forces, the non-uniform electric field gradient is induced by applying the electric voltage through the microchannel across a pair of asymmetric orifices, i.e., a small orifice and a large orifice on the opposite wall of the channel sides. The distribution of the gradient of the magnetic field near the edge of ferromagnet segments, the gradient of the non-uniform electric field in the vicinity of the asymmetric orifices, and the flow field were investigated. In this numerical simulation, the effects of the ferromagnet structure on the magnetic field, the flow rate, as well as the strength of the electric field on their combined magnetophoretic and dielectrophoretic behaviors and trajectories are systemically studied. The simulation results demonstrate the potential of both property- and size-based cell isolation in the microfluidic device by implementing magnetophoresis and dielectrophoresis.

## 1. Introduction

Cell sorting is a crucial step in the purification process and in biomedical research, including in food and pharmacy processing, environment monitoring, and clinical diagnostics, where detection of targeted cells with high accuracy and sensitivity is necessary [1]. The isolation of circulating tumor cells (CTCs) from blood specimens provides an essential method in diagnostic and therapeutic approaches, and shows great potential in improving cancer treatment [2,3]. It has been proven that the successful separation of CTCs enables cancer prognoses, response to anticancer drugs, and monitoring of the metastatic stage [4,5].

Generally, cell identification methods include fluorescent-activated, bead-based, and label-free cell sorting, which are utilized for the manipulation and recognition of cells [6]. Bonner et al. conducted flow cytometry for the separation of viable cells by using the fluorescence-activated cell sorter [7] and rapid and efficient purification procedures for human myoblasts were established [8]. By using nanomaterial-based structures, the label-free capture of CTCs and CTC clusters is constructed, providing novel perspectives to apply nanomaterials for the clinical capture and analysis of CTCs [9,10]. Yang et al. demonstrated the chemical-based capture of CTCs with a capture efficiency as high as 90% by using polyphenol tannic acid (TA)-functionalized films [11]. However, these methods still show various shortcomings, including complex pre-processes, bulky instruments, technical expertise, risky contamination of samples, the high cost of equipment, and safety concerns [2,12]. Compared with the above-mentioned techniques, microfluidics is becoming one of the most popular technologies to provide solutions for biological applications, such as manipulation and concentration of cells [3,13], bacteria [14], and 3D bioprinting [15]. Based on different physical mechanisms, various methods including dielectrophoresis [16,17,18,19], deterministic lateral displacement (DLD) [20,21,22,23], hydrodynamic lift [24], magnetophoresis [25], centrifugation [6], and acoustic [26] have been employed for cell separation in microfluidic chips, which benefit from bio-compatibility, low sample consumption, the tiny size of platform, and an easy process to carry out [27,28]. Moreover, due to the free of cell pre-label and decreasing cell damage, label-free cell sorting techniques in microfluidic devices are widely used for the separation of cells based on their specified physical differences [12]. Among these methods in a microfluidic chip, the active techniques, i.e., exerting external fields to induce forces on particles, such as dielectrophoresis and magnetophoresis, offer the possibility of cell sorting not only by size but also by type with a similar size [29].

Dielectrophoresis (DEP) provides great potential for cell discrimination and isolation for sample processing, sorting of biological cells [30,31], droplets [32,33] and particles [34,35,36,37,38,39,40]. Li et al. [41] utilized dielectrophoresis to separate live and heat-treated Listeria innocua cells. Gascoyne et al. [42] applied dielectrophoresis to isolate malaria-infected cells from blood. Moon et al. successfully separated human breast cancer cells (MCF-7) from a spiked blood cell sample by combining multi-orifice flow fractionation (MOFF) and dielectrophoresis [43]. Song et al. [44] utilized a continuous-flow microfluidic device based on the accumulation of multiple dielectrophoresis forces to sort stem cells and their differentiation progeny at different flow rates. Wang et al. proposed a novel microfluidic chip for the continuous separation of microalgae cells based on AC dielectrophoresis [45]. Vahey et al. demonstrated the separation of polystyrene beads based upon surface conductance as well as sorting non-viable from viable cells of the budding yeast Saccharomyces cerevisiae through dielectrophoresis [46]. Cao et al. [47] demonstrated highly effective enrichment of proteins by using nanoscale insulator-based dielectrophoresis (iDEP) integrated with Ag/SiO_2_ Nanorod Arrays. Kung et al. [48] utilized a tunnel dielectrophoresis (TDEP) mechanism for continuously tunable, sheathless, 3D, and single-stream microparticle and cell focusing in high-speed flows. However, in order to achieve a high separation resolution and enough throughput, high voltages are typically necessary to induce a strong DEP effect, which may induce Joule heating effects in the microchannel and limit their application for temperature-sensitive biological cells [49]. Magnetophoresis (MAP) is always utilized as one of the most efficient methods for bulk cell separation [50,51]. Jung et al. [52] achieved cell separation through a magnetophoretic device integrated with slanted ridge arrays in a microfluidic channel. A total of 91.68 ± 2.18% of Escherichia coli bound with magnetic nanoparticles are successfully separated from undiluted whole blood. Karle et al. [53] demonstrated continuous DNA extraction from cell lysate on a microfluidic chip based on phase-transfer magnetophoresis for the first time. Han et al. [54] utilized a continuous magnetophoretic microseparator to separate white and red blood cells from whole blood based on their native magnetic properties. Zhu et al. [55] demonstrated a novel separation method combining positive and negative magnetophoreses based on ferrofluids to separate mixtures of particles with different magnetic properties. Pamme et al. [51] separated mouse macrophages and HeLa cells successfully in a microfluidic magnetic separation device. James et al. [56] proposed sorting spermatozoa by magnetophoresis and shear flow to reduce the percentage of abnormal sperm cells for assisted reproduction. Therefore, the operation of the integrated dielectrophoretic and magnetophoretic microfluidic chip for the manipulation of biological cells provides a magnetophoretic pre-enrichment before the dielectrophoretic stage and enhances the sensitivity and efficiency of cell manipulation.

In this study, a novel method combining dielectrophoresis and magnetophoresis is proposed for the separation of CTCs from cell pools. The microfluidic chip used in the simulation achieved cell isolation by passing through the multiple sorting regions, i.e., the magnetophoretic pre-screening and the dielectrophoretic isolation consequently. The effect of the flow rate, the AC electric potential and the ferromagnet structure on the separation were investigated. By adjusting the applied magnetic field and the non-uniform electric field, the separation of two types of CTCs, i.e., MDA-231 and HT-29, from the mixture with platelets and red blood cells is numerically examined.

## 2. Materials and Methods

### 2.1. Separation Mechanism

**Figure 1:**
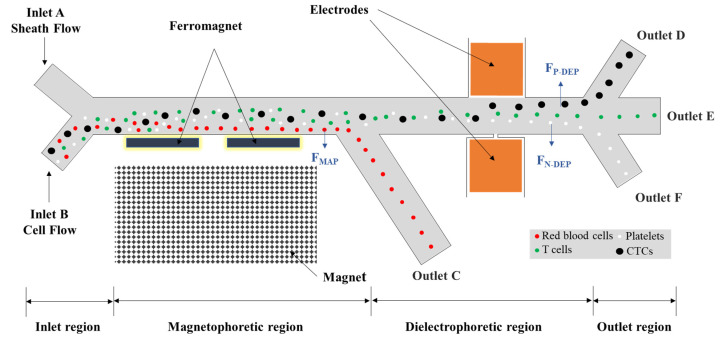
The schematic illustration of the microfluidic channel.

The microchannel is composed of a sample inlet region, a magnetophoretic and dielectrophoretic sorting region, and an outlet sample connection region. When passing through the magnetophoretic region, the trajectories of magnetic spheres shift based on their magnetic susceptibilities. Afterwards, the samples are separated based on their different dielectrophoretic behaviors. To determine the label-free separation of multiple types of different cells in the microfluidic chip, the isolation of red blood cells, platelets, T cells, and two types of CTCs, i.e., HT-29 and MDA-231, was conducted in a microfluidic chip. Figure 1 shows the illustration of CTC sorting from blood cells by the combination of dielectrophoretic and magnetophoretic methods in the microchannel. The mixed cell samples flow into the main horizontal channel, where the cells are forced to move close to the sidewall of the magnet by the dominant suspending medium from the sheath flow. The red blood cells are discriminated in the magnetophoretic region and collected in outlet C. Then, the rest cells continue entering into the dielectrophoretic region. To induce the dielectrophoretic forces, the non-uniform electric field is generated through a pair of asymmetric orifices, i.e., a small orifice on one side of the microchannel wall and a large one on the opposite channel wall. Based on their distinct dielectric property and size, the remaining CTC cells, T cells, and platelets are separated and move into outlet channels (D, E, and F), respectively.

Magnetophoresis refers to the movement of particles in a magnetic field [57]. If a particle is suspended in a magnetic fluid medium with a relative magnetic permeability of $u_{m}$, the magnetophoretic force acting on a spherical particle with a radius of r can be expressed as [58]

(1) $$F_{MAP}=\frac{4}{3}u_{m}u_{0}\pi r^{3}K\left(H\cdot \nabla \right)H$$

where $u_{0}$ is vacuum permeability and $u_{0}$ = 4π × 10^−7^, H is the strength of the applied magnetic field, and K is described as

(2) $$K=3\left(\chi_{p}-\chi_{m}\right)/ [\left(\chi_{p}-\chi_{m}\right)+3\left(\chi_{m}+1\right)]$$

where $\chi_{p}$ is the susceptibility of particles and $\chi_{P}=u_{P}-1$, $u_{p}$ is the relative permeability of particles, and $\chi_{m}$ is magnetic susceptibility of magnetic fluid medium. It can be seen from Equation (2) that the direction of the magnetophoretic force is determined by the magnetic susceptibility of the particle and the surrounding medium. If the magnetic susceptibility of the cells is greater than that of the surrounding medium, they will be subjected to the positive magnetophoretic force. On the contrary, the negative magnetophoretic force would be acting on the cells.

Dielectrophoresis describes the movement of dielectric particles in a non-uniform electrical field due to the polarization difference between the particle and the suspending medium. The general expression for the dielectrophoretic force acting on a spherical particle is expressed as [59]

**Figure 2:**
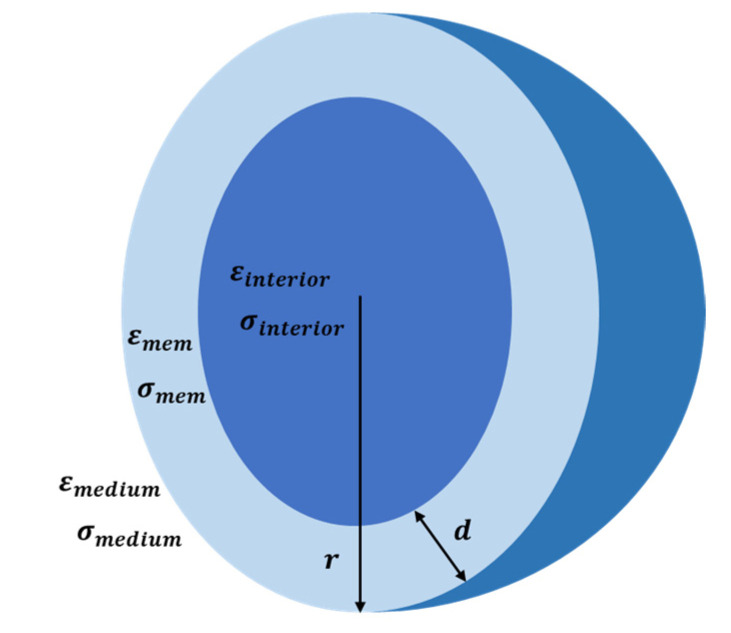
Schematic of a spherical cell with a single shell [59,62].

(3) FDEP=2πεm r3 RefCM∇E2

where r is the radius of the spherical particle, $\varepsilon_{m}$ is the electric permittivity of the suspending medium, $\nabla {\left|E\right|}^{2}$ is the gradient of the electric field squared, and RefCM is the real part of the complex Clausius–Mossotti (CM) factor, which describes the relative polarizability of the particle and of the suspension medium, which is a function of geometry and frequency. For cells, the CM factor utilizing the double-shell model (shown in Figure 2) is defined by [13,59,60,61]

(4) $$f_{CM}=\left(\varepsilon_{cell}^{*}-\varepsilon_{m}^{*}\right)/\left(\varepsilon_{cell}^{*}+2\varepsilon_{m}^{*}\right)$$

(5) $$\varepsilon_{cell}^{*}=\varepsilon_{membrane}^{*}\frac{{ [\frac{r}{r-d}]}^{3}+2 [\frac{\varepsilon_{interior}^{*}-\varepsilon_{membrane}^{*}}{\varepsilon_{interior}^{*}+2\varepsilon_{membrane}^{*}}]}{{ [\frac{r}{r-d}]}^{3}- [\frac{\varepsilon_{interior}^{*}-\varepsilon_{membrane}^{*}}{\varepsilon_{interior}^{*}+2\varepsilon_{membrane}^{*}}]}$$

where $\varepsilon_{c}^{*}$ and $\varepsilon_{m}^{*}$ are the complex permittivity of cells and media, $\varepsilon^{*}=\varepsilon -\left(j\sigma /\omega \right)$ represents the complex permittivity, ε indicates the permittivity of the cell and solution, σ is the corresponding electrical conductivity, $j=\sqrt{-1}$, ω=2πf expresses the angular frequency of the applied electric field, and f is the ordinary frequency.

It can be indicated from Equation (3) that the magnitude of the DEP force is proportional to the cell size ($\;r^{3}$) and the gradient of the non-uniform electric field ($\nabla {\left|E\right|}^{2}$), while the direction of the DEP trajectories of the cells is determined by the sign of $f_{CM}$. If the polarizability of the cell is greater than that of the medium, i.e., a positive value of CM factor, the cells will be driven by the positive dielectrophoretic forces (p-DEP) moving towards the stronger non-uniform electric field area. One the other hand, a negative value of CM factor refers to the negative dielectrophoretic effects (n-DEP) and the cells will move towards the weaker non-uniform electric field region. Based on various sizes and the unique property of cells, they have different trajectories and move into different individual outlet collections in the microchannel.

### 2.2. Physical and Mathematical Models

**Figure 3:**
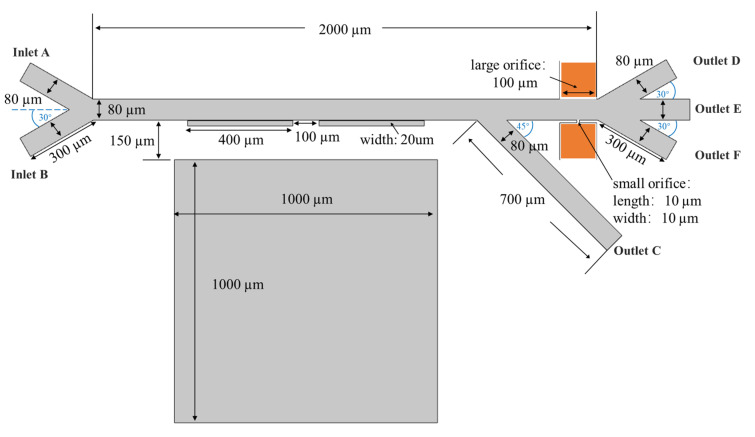
Schematic illustration of the integrated microchannel.

The top view of the geometrical illustration of the integrated dielectrophoretic and magnetophoretic microfluidic chip is shown in Figure 3. This microchannel includes a main channel with a length of 2000 μm and a width of 80 μm together with six branches. The focusing sheath flow inlet (A) and sample inlet (B) channels are 300 μm in length and 80 μm in width, the magnetophoretic separation region is composed of a pair of ferromagnets with a width of 400 μm and a length of 20 μm and a permanent magnet with both length and width of 1000 μm, the outlet (C) channel connecting to the magnetophoretic separation region has a length of 700 μm and a width of 80 wide, a pair of asymmetric orifices are embedded with external micro-electrodes, where the small orifice has both length and width of 10 μm and the large orifice has a width of 100 μm, and three outlet channels (D, E, and F) next to the dielectrophoretic separation region have a length of 300 μm and a width of 80 μm. Driven by the pressure flow, and the sheath flow from channel A, the mixed cell sample flows from channel B into the horizontal main channel. The cells are forced to flow closely to the ferromagnets by the focusing stream to experience the magnetophoretic effects and their trajectories shift. For the cells experiencing positive magnetophoretic forces, they will be attracted towards the ferromagnets and move into outlet C. However, the negative magnetophoretic forces exerting on the cells lead the repellent away from the ferromagnets and then they continue entering into the dielectrophoretic sorting area. For particles with a $f_{\mathrm{CM}}>$ 0, they will experience a positive DEP force and be attracted towards the small orifice, and move into outlet F. However, for particles with a $f_{\mathrm{CM}}<$ 0, they will be repelled toward the large orifice and move into outlet D and E based on their sizes, respectively.

#### 2.2.1. The Flow Field

The liquid flow in the microchannel is considered as incompressible laminar flow and the flow field is governed by the Naiver–Stokes equation

(6) $$\rho [\frac{\partial \vec{u}}{\partial t}+\vec{u}\cdot \nabla \vec{u}]=-\nabla P+\mu \nabla^{2}\vec{u}$$

and the continuity equation is described as

(7) $$\rho \nabla \vec{u}=0$$

where $\vec{u}$ illustrates the velocity vector, μ and ρ are the viscosity and density of the medium, and ∇P is the gradient of pressure. As the flow velocity inside the microchannel is relatively low, the inertia term $\vec{u}\cdot \nabla \vec{u}$ in Equation (6) is negligible. Furthermore, the flow field does not change over time in the steady flow, the term of $\frac{\partial \vec{u}}{\partial t}$ can also be neglected. The velocity of the inlet and outlet microchannels is set with determined values

(8) $$u=u_{1},\text{ at the inlet}$$

(9) P=0, at the outlet

(10) $$\vec{u}=0,\text{ at the channel walls}$$

#### 2.2.2. The Magnetic Field

The magnetic field in the microchannel is governed by Maxwell’s equation

(11) ∇×H=J

(12) $$H=-\nabla V_{m}$$

where H represents the magnetic field intensity and $V_{m}$ is the magnetic scalar potential. According to Gauss’ law, the magnetic flux density  B is expressed as

(13) ∇⋅B=0

The magnetization model of the constitutive relation B–H of the magnet is set as the residual flux density, which is defined by

(14) $$B=\mu_{0}\mu_{rec}H+B_{r}$$

(15) $$B_{r}=\|B_{r}\|\frac{e}{\left\|e\right\|}$$

where $B_{r}$ is the residual magnetic flux density, e represents the direction of the residual magnetic flux, $\mu_{rec}$ is the recovery permeability, and $\mu_{0}$ is vacuum permeability. The other areas except the permanent magnet are described as

(16) $$B=\mu_{0}\mu_{r}H$$

where $\mu_{r}$ represents the relative permeability.

#### 2.2.3. The Electric Field

As shown in Figure 3, the electric potential is applied across the microchannel through a pair of asymmetric orifices. The distribution of the applied electric field φ is governed by Laplace’s equation

(17) $$\nabla^{2}\varphi =0$$

and the non-conducting boundary condition on the microchannel walls except the asymmetric orifices region, and the specific electric potential applied to the external micro-electrodes are expressed as

(18) $$\varphi =V_{1},\;\mathrm{at}\;\mathrm{the}\;\mathrm{small}\;\mathrm{orifice}$$

(19) φ=0, at the large orifice

(20) $$\hat{n}\cdot J=0,\;\mathrm{at}\;\mathrm{the}\;\mathrm{channel}\;\mathrm{walls}$$

where $\hat{n}$ is the unit normal vector and J=∇φ is the electric current density.

#### 2.2.4. Particle Tracing

In order to visualize the trajectories of various cells moving in the microchannel, the cell movement is considered to be coupled with the electric field, the magnetic field and the flow field. The motion of cells is governed by Newton’s second law [63,64,65]

(21) $$m_{c}\frac{d\vec{v}}{dt}=\vec{F_{t}}$$

where $m_{c}$ and $\vec{v}$ are the mass and velocity of the cell, and $\vec{F_{t}}$ represents the net force exerting on the cell, including the viscous drag force, the MAP force, and the DEP force.

Due to the liquid viscosity, the drag forces act on the cells when they are flowing in the liquid. For the laminar flow, the drag force exerting on the cells is known as Stokes drag law, which is defined by

(22) $${\vec{F}}_{drag}=\frac{1}{\tau_{c}}m_{c}\left(\vec{u}-\vec{v}\right)$$

(23) $$\tau_{c}=\frac{2\rho_{c}r^{2}}{9\mu }$$

where  μ is the dynamic viscosity of the liquid flow and $\rho_{c}$ is the density of the cell.

The dielectrophoretic force and magnetophoretic force acting on the cell are given by

(24) FDEP=2πεm r3 RefCM∇E2

(25) $$F_{MAP}=\frac{4}{3}u_{m}u_{0}\pi r^{3}K\left(H\cdot \nabla \right)H$$

### 2.3. Numerical Simulation

In this study, commercial software COMSOL 5.5 was utilized to solve the above-mentioned equations and boundary conditions for the numerical simulation of the isolation of CTC cells from red blood cells, platelets, and T cells. Firstly, the flow field, the magnetic field and the electric field were computed using the stationary solver. Then, the mixture of cells was introduced into the microchannel using the particle tracing module. The trajectories of the mixed cells with the MAP and DEP effects were solved in the computation. To investigate the various effects of the ferromagnet structure, the flow rate, and the strength and the frequency of the electric field on the trajectories of cells, extensive numerical simulations were conducted and analyzed. The values of parameters utilized in the simulation are shown in Table 1.

To discuss the separation resolution, different red blood cells (RBC), T lymphocytes (T cells), platelets, and two types of circulating tumor cells (CTCs), i.e., HT-29 (human colon cancer cells) and MDA-231 (human breast cancer cell), were used and the corresponding values of parameters are shown in Table 2.

## 3. Results and Discussion

### 3.1. The Effect of the Ferromagnet Structure on the Magnetic Field

**Figure 4:**
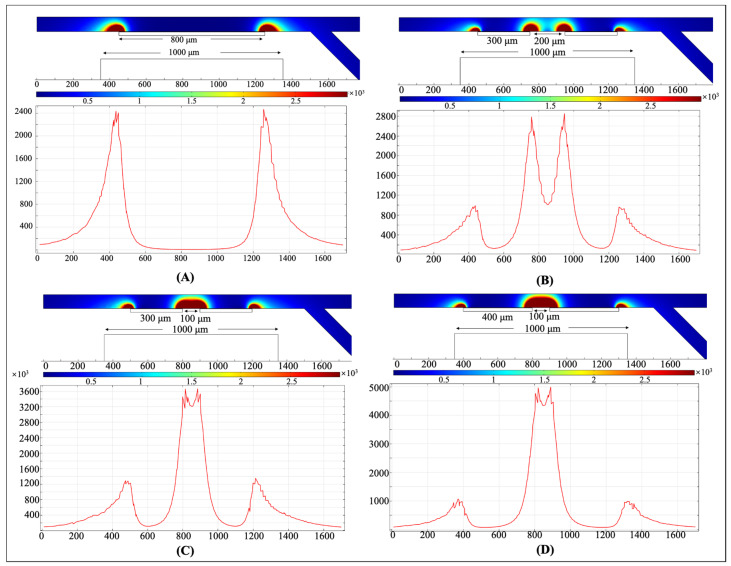
Numerical simulations of the effect of the ferromagnet structure on the gradient of the magnetic field. (**A**) Single-segment ferromagnet with a length of 800 μm; double-segment ferromagnets with (**B**) a length of 300 μm and a distance of 200 μm, (**C**) a length of 300 μm and a distance of 200 μm, and (**D**) a length of 400 μm and a distance of 100 μm. The length of the permanent magnet is 1000 μm. The residual magnetic flux density is 1 T. The stronger magnetic field is depicted by the darker red color and higher magnitude.

To discuss the effect of the ferromagnet structure on the gradient of the magnetic field, extensive numerical simulations have been conducted and Figure 4 show some examples of the values of the magnetic field gradient obtained for the ferromagnet combinations with different pairs of ferromagnet segment, and double-segment ferromagnets with two different lengths and distances at a given magnetic field.

As we know, a higher gradient of the magnetic field means stronger MAP forces. It can be inferred from Figure 4A,B that the structure with double-segment ferromagnets induces higher values of the magnetic field (depicted by the darker red color) near the segment edge. By comparing Figure 4B,C, once can clearly find that the region of the high gradient of the magnetic field is larger in the structure with the shorter distance between two ferromagnet segments (Figure 4C). For example, the semicircle of the darker red color has a radius of approximately 40 μm in Figure 4B, while the semicircle of the darker red color has a radius of approximately 70 μm in Figure 4C. This is because, when the distance between the ferromagnet segments is reduced, the magnetic field is more compressed near the segments. Therefore, one can clearly see that the magnitude of the magnetic field gradient is higher and the high gradient of the magnetic field area is larger in the structure with shorter distance between two ferromagnet segments (Figure 4C). Therefore, the structure with multi-segment ferromagnets and a relatively shorter distance between the ferromagnet segments can lead to a stronger MAP force on the cells, resulting in a high separation resolution. Furthermore, it can be expected that increasing the length of ferromagnet segments will increase the value of the maximum magnitude of the magnetic field gradient and the corresponding high gradient of the magnetic field region significantly, as shown in Figure 4D.

### 3.2. The Effect of the Flow Rate

**Figure 5:**
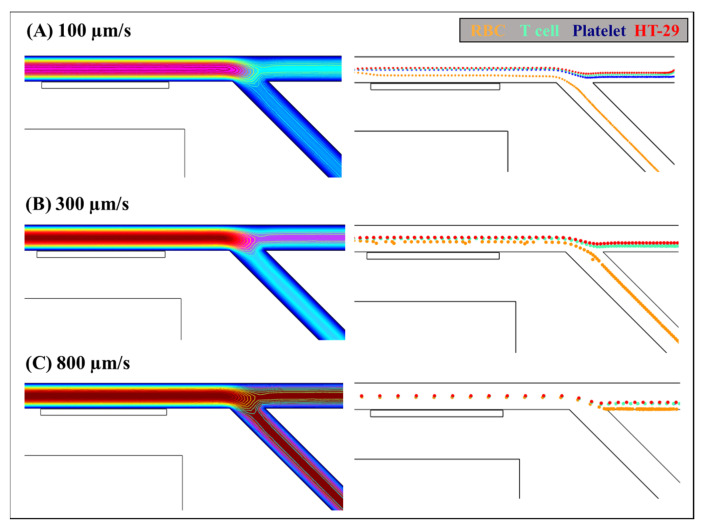
The effect of the flow rate on the separation of RBCs, T cells, platelets, and HT-29 by the integrated MAP-DEP microchannel. The dependence of the cell trajectories on pressure-driven flow with cell velocity of (**A**) 100, (**B**) 300, and (**C**) 800 μm/s. The width of the channel is 80 μm and the residual magnetic flux density is 1T.

To examine the effect of pressure-driven flow, three different velocities of cells mixture, i.e., 100, 300, 800 μm/s, were tested for the separation of RBCs, T cell, platelet, and HT-29. In these tests, the residual flux density of permanent magnet is applied 1 T. The trajectory shifts of the mixed cell sample with different flow rates are demonstrated in Figure 5.

It can be illustrated from Figure 5A that the RBCs experienced positive magnetophoretic forces and were attracted into outlet channel C, and the rest mixtures were repelled away from the double-segment ferromagnet by the negative magnetophoretic effects and continued flowing into the dielectrophoretic sorting region at the cell velocity of 100 μm/s. It is easy to understand that when the velocity of the cells increases, they will move faster and pass over the vicinity of the ferromagnet. The time period of the cells undergoing the MAP forces will decrease. As shown in Figure 5B, when the flow velocity was increased to 300 μm/s, the time period of MAP effects acting on the cells is shortened, and the trajectory differences between the RBCs and the rest cells is reduced after passing through the vicinity of the ferromagnet segments. Then, most of RBCs were still isolated into the outlet channel C, but some of them moved together with the rest mixture flowing to the outlet channel. However, a further increase in the cell velocity excessively shortened the acting time of the MAP forces exerting on the cells, and the RBCs could not be separated from the mixed cells into individual outlet channels. As shown in Figure 5C, when the velocity of cells increased to 100 μm/s, the RBCs cannot experience strong enough positive MAP forces and flow passing over the outlet channel C, which were driven together with the rest cells into the next DEP sorting region.

### 3.3. The Effect of the Applied Voltage

**Figure 6:**
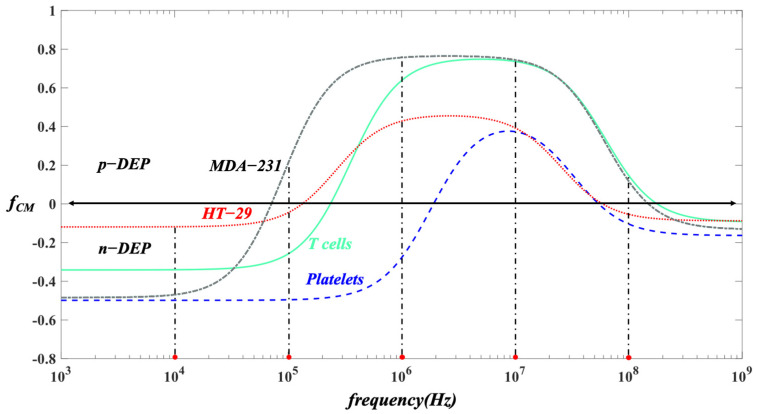
Prediction of *f_CM_* values for HT-29 (red dash line), T cells (solid green line), and platelets (blue dash line) varying with the electric field frequency.

**Figure 7:**
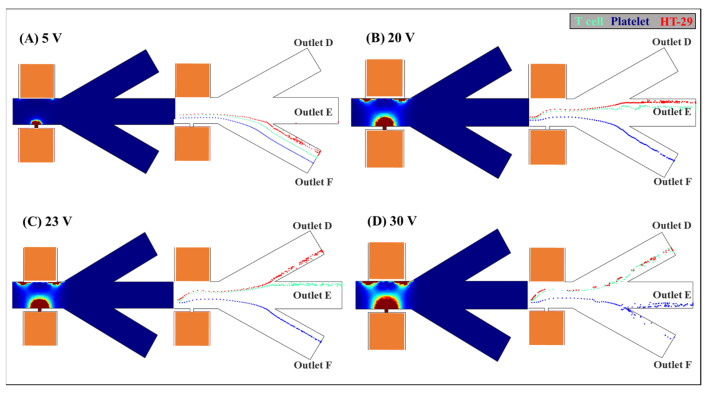
Numerical simulations of the distribution of the electric field gradient
$$\left(\nabla {\left|E\right|}^{2}\right)$$
and the effect of the applied electric field on the separation of the T cells, platelets, and HT-29 by the integrated MAP-DEP microchannel. The dependence of the cell trajectories on the applied voltage at the small orifice: (**A**) 5, (**B**) 20, (**C**) 23, and (**D**) 30 V. The large orifice is grounded. The frequency of the electric field is 104 Hz and the velocity of the cells is 300 μm/s. The width of the main channel is 80 μm. The width and length of the small orifice are 10 μm, and the width of the large orifice is 100 μm.

After passing through the magnetophoretic isolation area, the RBCs were sorted from the cells mixture by positive magnetophoretic effects and moved into the outlet channel C. The rest of the mixed cells continued flowing into the dielectrophoretic separation region. To determine the effect of applied voltage on the DEP separation of T cells, platelets, and HT-29, different voltages from 5 to 30 V at small orifice were studied. The large orifice is grounded. The frequency of the electric field is 10^4^ Hz (i.e., the cells experience negative DEP effects as shown in Figure 6) and the velocity of the cells is 300 μm/s. The width of the main channel is 80 μm. The width and length of the small orifice are 10 μm, and the width of the large orifice is 100 μm. In these simulations, the trajectory differences of T cells, platelets, and HT-29 under different applied voltages from 5 to 30 V are shown in Figure 7.

It should be known that according to Equation (3), the DEP force is proportional to the gradient of the non-uniform electric field ($\nabla {\left|E\right|}^{2}$). As the applied voltages increase, the DEP forces will be enhanced. It can be inferred from Figure 7 that an increase in the applied voltage leads to larger values of the maximum $\nabla {\left|E\right|}^{2}$ (i.e., larger semicircle of the darker red color) and wider high ($\nabla {\left|E\right|}^{2}$) areas, as shown in Figure 7D. Furthermore, it can be indicated from Equation (3) that the magnitude of the DEP force acting on the HT-29 (6.6 μm in radius) is approximately 7-fold larger than that on the T cells (3.4 μm in radius), and the magnitude of the DEP force acting on T cells (3.4 μm in radius) is 54-fold larger than that on the platelets (0.9 μm in radius). As shown in Figure 7A, under 5 volts, the DEP forces is relatively weak, and thus shows non-apparent trajectory differences between the cells mixture after passing through the vicinity of the small orifice, and they all moved into the outlet channel F. When the applied voltage was increased to 20 volts, as shown in Figure 7B, the larger DEP forces led to a significant shift difference between the small platelets and the large HT-29 and T cells after passing over the small orifice. Although they were separated from the platelets, the DEP forces acting on the large HT-29 and T cells were still not large enough, both of which moved into the outlet channel E. It can be illustrated from Figure 7C, when the applied voltage was increased to 23 volts, the stronger DEP effects resulted in greater trajectory variation for the larger HT-29, which were isolated from T cells and moved into individual outlet channels D and E. The separation of T cell, platelet, and HT-29 with different sizes was achieved. Then, further increasing the applied voltage may induce overly strong DEP effects and cannot achieve the separation of the small and large cells into distinct outlet channels as desired. As shown in Figure 7D, the smallest platelets were further pushed by the DEP forces and part of them moved into outlet channel E, while the larger HT-20 and T cells were repelled away and moved together into the outlet channel D.

### 3.4. Isolation of CTCs

To examine the separation sensitivity of the proposed MAP-DEP separation method, two types of CTCs, i.e., MDA-231 and HT-29, were mixed with RBCs and platelets to determine the numerical simulation. The voltage of 11 V is applied at the small orifice and the large orifice is grounded. The residual magnetic flux density is 1 T. The frequency of the electric field is 10^4^ Hz (i.e., the cells experience negative DEP effects as shown in Figure 6) and the velocity of the cells is 300 μm/s. The width of the main channel is 80 μm. The width and length of the small orifice are 10 μm, and the width of the large orifice is 100 μm.

**Figure 8:**
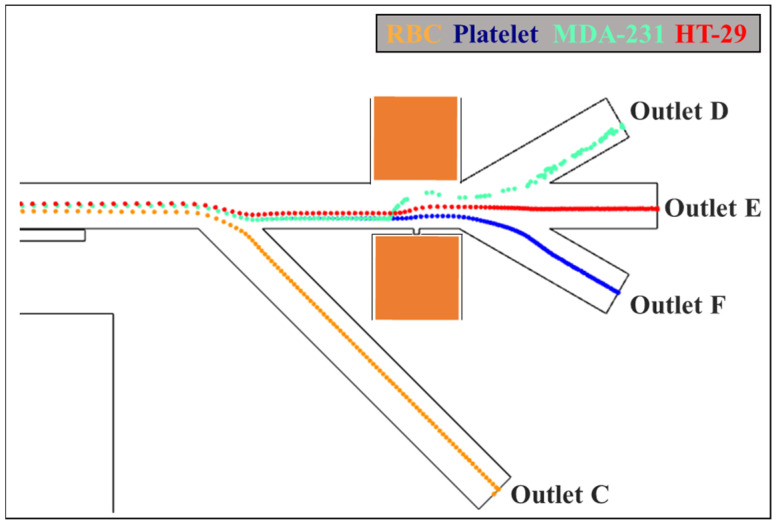
Numerical simulation of the separation of RBCs, MDA-231, HT-29, and platelets. The voltage of 11 V is applied at the small orifice and the large orifice is grounded. The residual magnetic flux density is 1 T. The frequency of the electric field is 10^4^ Hz and the velocity of the cells is 300 μm/s. The width of the main channel is 80 μm. The width and length of the small orifice are 10 μm, and the width of the large orifice is 100 μm.

It can be illustrated from Figure 8 that the RBCs experienced positive magnetophoretic forces and were attracted into outlet channel C, and the rest mixture of MDA-231, HT-29, and platelets were repelled away from the double-segment ferromagnet by the negative magnetophoretic effects and moved into the dielectrophoretic sorting region. By applying a specific electric field frequency, the isolated dependent on different dielectrophoretic behaviors as a function of cell radius. The smallest platelets experienced relatively weak negative DEP effects and moved closely to the vicinity of the small orifice into the outlet channel F. While two different types of CTCs, i.e., MDA-231, HT-29, were separated based on their different size, the larger, MDA-231, were repelled away by the stronger negative DEP forces and flowed into outlet channel D, and HT-29 were pushed into outlet channel E. This indicates that the proposed dielectrophoretic and magnetophoretic isolation method can be used for the separation of various types of CTCs, as demonstrated in Figure 8.

## 4. Conclusions

The manipulation and isolation of CTCs by using the integrated DEP and MAP method in a pressure-driven flow were numerically studied. To induce sufficient MAP effects, the strong gradient of the magnetic field was produced by using multiple pairs of ferromagnet segments. By using the ferromagnet structure with double segments and a relatively shorter distance in between, the strong gradient of the magnetic field, i.e., sufficiently large MAP forces acting on the cells, was generated, leading to a high separation resolution. In order to generate strong DEP forces, the non-uniform electric field gradient was induced by applying the electric voltage through the microchannel across a pair of asymmetric orifices, i.e., a small orifice and a large orifice on the opposite wall of the channel sides. The distribution of the gradient of the magnetic field near the edge of ferromagnet segments, the gradient of the non-uniform electric field in the vicinity of the asymmetric orifices, and the flow field were investigated. To examine the different MAP behaviors of the cells mixture, the gradient of the magnetic field in terms of the ferromagnet structures was studied. Furthermore, a suitable flow rate was found necessary for the separation of mixed cells when passing over the MAP isolation region. If we further increase the velocity of the cells, the action time of MAP forces on the cells will be excessively shortened and they will move out of the MAP sorting area. By selecting a specific electric field frequency, the rest mixture of CTCs continues flowing into the DEP separation region and is sorted based on distinct properties and different sizes. In this numerical simulation, the effects of the ferromagnet structure on the magnetic field, the flow rate, as well as the strength of the electric field on their combined magnetophoretic and dielectrophoretic behaviors and trajectories were systemically studied, proving this proposed integrated MAP-DEP method can be used for the isolation of CTCs by size and dielectric properties.

## Figures and Tables

**Table 1:** Values of parameters used in the simulation.

Parameters	Values
$\mathrm{Dielectric}\;\mathrm{constant}\;\mathrm{of}\;\mathrm{buffer}\;\mathrm{liquid}\;\mathrm{phase},\;\varepsilon_{m}$	80
$\mathrm{Permittivity}\;\mathrm{of}\;\mathrm{vacuum},\;\varepsilon_{0}$ (F m^−1^)	8.85 × 10^−12^
$\mathrm{Vacuum}\;\mathrm{permeability},\;\mu_{0}$ (N A^−2^)	4π × 10^−7^
$\mathrm{Density}\;\mathrm{of}\;\mathrm{buffer},\;\rho_{b}$ (kg m^−3^)	1000
$\mathrm{Dynamic}\;\mathrm{viscosity}\;\mathrm{of}\;\mathrm{buffer},\;\mu_{b}$ (Pa·s)	1 × 10^−3^
Electrical conductivity of buffer (mS m^−1^)	55
$\mathrm{Density}\;\mathrm{of}\;\mathrm{particles},\;\rho_{p}$ (kg m^−3^)	1050
$\mathrm{Magnetic}\;\mathrm{susceptibility}\;\mathrm{of}\;\mathrm{buffer},\;\chi_{m}$ (kg m^3^)	−9 × 10^−6^
$\mathrm{Residual}\;\mathrm{flux}\;\mathrm{density}\;\mathrm{of}\;\mathrm{permanent}\;\mathrm{magnet},\;\|B_{r}\|$ (T)	1
Length of main channel (μm)	2000
Length of microchannel A, B, D, E and F (μm)	300
Length of microchannel C (μm)	700
Width of the whole microchannel (μm)	80

**Table 2:** Physical-chemical properties of the cells used in the simulation [29,62,66,67,68,69,70].

Property	RBC	T cells	Title 2	Title 3	Title 4
interior conductivity (S/m)	0.31	0.65	0.203	0.62	0.25
membrane conductivity (S/m)	1 × 10^−6^	27.4 × 10^−6^	34.82 × 10^−6^	0.9 × 10^−6^	1 × 10^−6^
interior dielectric constant	59	60	61.14	52	50
membrane dielectric constant	4.4	11.1	6.01	14.69	6
magnetic susceptibility (m^3^/kg)	−3.9 × 10^−6^	−9.9 × 10^−6^	−9.5 × 10^−6^	−9.5 × 10^−6^	−9.2 × 10^−6^
radius (μm)	3	3.4	6.6	9	0.9
membrane thickness (nm)	9	7	5	7	8

## Data Availability

Not applicable.
